# The GOLD-PCP Study: Clinician Insights on Person-Centric Packaging Design of a Triple Fixed-Dose Combination in Type 2 Diabetes Care

**DOI:** 10.7759/cureus.95473

**Published:** 2025-10-26

**Authors:** Chitra Selvan, Lakshmi Nagendra, Parth Jethwani, Sachin Mittal, Sanjay Kalra, Sunetra Mondal, Tejal Lathia, Amit Gupta, Smriti Gadia, Thamburaj Anthuvan

**Affiliations:** 1 Endocrinology, M. S. Ramaiah Medical College, Bengaluru, IND; 2 Endocrinology, JSS Medical College, Mysuru, IND; 3 Endocrinology and Metabolism, Kota Heart Institute, Kota, IND; 4 Endocrinology, Care Plus Clinic, Chandigarh, IND; 5 Endocrinology, Bharti Hospital, Karnal, IND; 6 Endocrinology, Nil Ratan Sircar Medical College and Hospital, Kolkata, IND; 7 Endocrinology, Apollo Hospitals, Navi Mumbai, IND; 8 Scientific Services, USV Private Limited, Mumbai, IND; 9 Sales and Marketing, USV Private Limited, Mumbai, IND

**Keywords:** dapagliflozin, diabetes therapy, fixed-dose combination, glimepiride, medication adherence, metformin, patient-centered care, person-centric packaging, real-world evidence

## Abstract

Introduction: Medication adherence remains a significant challenge in managing type 2 diabetes, particularly for patients on long-term polypharmacy. Fixed-dose combinations (FDCs) may improve adherence by reducing pill burden, while person-centric packaging (PCP) aims to enhance ease of use and identification. The GOLD-PCP study explored clinician perceptions of PCP’s impact on adherence and treatment management in diabetes care.

Methods: A cross-sectional survey was conducted with 262 clinicians from India who had been prescribing this FDC with PCP for at least six months. Clinician perceptions were assessed through a validated 12-item questionnaire administered following structured round-table meetings. Data were analyzed using descriptive statistics and Pearson’s correlation in IBM Corp. Released 2021. IBM SPSS Statistics for Windows, Version 27. Armonk, NY: IBM Corp.

Results: Clinicians perceived improvements in patient adherence (83.2%), therapy simplification (82.1%), and medication identification (82.0%). Most (80.9%) believed the FDC helped reduce pill burden. The floral PCP design was perceived as helpful in increasing patient preference (78.6%), reducing pharmacy substitution (80.1%), and lowering missed doses (80.2%). A strong correlation was observed between perceived pill burden reduction and perceived adherence improvement (r = 0.883, p < 0.001).

Limitations: This study assessed clinician perceptions only and did not include direct patient-level adherence measurements, objective adherence data, or patient-reported outcomes.

Conclusion: Clinicians perceived PCP as beneficial for adherence support and therapy management in type 2 diabetes. However, further patient-centered studies using objective adherence metrics are needed to validate these perceptions and determine causal relationships between packaging design and adherence outcomes.

## Introduction

Medication adherence is a crucial part of chronic disease care, especially in type 2 diabetes mellitus (T2DM), where patients often need multiple medications daily. As treatment becomes more complex, patients may struggle to stay on track, leading to poor blood sugar control, a higher risk of complications, and increased healthcare costs [1]. Studies show that as the number of pills increases, patient adherence declines, making disease management more difficult [2]. Fixed-dose combinations (FDCs) help simplify treatment by reducing the number of pills, which, in turn, improves adherence [3]. However, even with FDCs, certain real-world barriers to adherence remain unaddressed.

One such barrier is the design of medication packaging. Poorly designed packaging can lead to confusion, errors in dosing, or missed doses, especially in elderly patients or those taking multiple medicines. Similar-looking pills, unclear labels, and lack of structured instructions can all contribute to non-adherence [4,5]. Recent research highlights that improving packaging, through clear labeling, color coding, or visual cues, can make it easier for patients to recognize and correctly use their medications [6].

To address these issues, person-centric packaging (PCP) has been developed as a patient-friendly design approach that supports easier identification and regular use of medicines. PCP often include features like clear instructions, structured layouts, and distinctive visuals that help reduce errors and boost confidence in treatment. While PCP has been proposed as a helpful tool for improving adherence, especially when used with FDCs, real-world data from clinical practice remains limited.

This study aimed to evaluate clinicians’ perceptions of the impact of PCP on adherence, therapy simplification, and patient preference in the case of a triple FDC containing dapagliflozin, glimepiride, and metformin. By capturing clinician experiences and perceptions, this study aims to generate practical, real-world insights into how packaging design can enhance the effectiveness of FDC-based diabetes care.

## Materials and methods

Study design

This cross-sectional study assessed clinician perceptions of a triple FDC of dapagliflozin, glimepiride, and metformin provided in PCP. Clinicians were recruited using convenience sampling from diverse regions in India. Eligible participants were licensed practitioners with at least six months of experience prescribing the FDC with PCP. The study was conducted between November and December 2024. Of 350 clinicians approached, 262 participated, representing northern, southern, western, and eastern India. Informed consent was obtained within the survey platform, ensuring ethical compliance and structured participation.

Pre-survey educational session

Prior to survey administration, 60-minute round-table meetings were conducted to provide standardized information about the FDC and its PCP features. While these sessions ensured consistent product knowledge, they may have introduced information bias by priming participants toward more favorable perceptions of the intervention. To reduce this, the survey was administered from November 2024 to January 2025, and clinicians were instructed to respond based on their independent experience.

Questionnaire

The survey questionnaire was developed in four structured phases: questionnaire development, validity assessment, pilot testing, and reliability assessment. The initial draft underwent multiple rounds of qualitative and quantitative content validation by three independent experts. A pilot study was conducted among eligible clinicians, refining the questionnaire from 17 to 12 questions based on feedback. A 12-item questionnaire was developed and validated through expert review (five endocrinologists and three clinical pharmacists), followed by pilot testing with 20 clinicians. The final validated questionnaire comprised two sections covering key aspects of medication adherence, therapy simplification, patient preference, cost-effectiveness, and medication identification (Appendix Table 1). To ensure methodological rigor, internal consistency was evaluated using Cronbach’s alpha, achieving an acceptable reliability score. Responses were recorded on a 5-point Likert scale (1 = strongly disagree to 5 = strongly agree). The final study protocol and questionnaire were reviewed and approved by two members of the steering committee. The validation process adhered to best practices for scale development in health and behavioral research, ensuring content relevance, clarity, and reliability [7].

Survey and data collection

A convenience sampling approach was used to select participants. The structured questionnaire was distributed via a unique Google (California, USA) survey link sent to each clinician’s registered email. The survey was configured to ensure complete data capture, prevent duplicate entries, and eliminate missing responses. Before dissemination, all participants attended an online briefing session, conducted by the steering committee, to standardize understanding of study objectives and procedures. Responses were exclusively collected through the Google platform to maintain data consistency and security. Real-time data monitoring was implemented to ensure adherence to the study protocol, with automated system checks flagging incomplete or inconsistent responses for review. Responses not meeting quality standards were excluded from the final analysis.

Sample size justification and power consideration

The sample size of 262 clinicians was determined to ensure adequate statistical power for correlation analysis based on expected effect sizes from prior literature on medication adherence interventions. An a priori power analysis using G*Power Ver. 3.1 Heinrich-Heine-Universität Düsseldorf, Düsseldorf, Germany, indicated that a minimum of 138 participants would be required to detect a moderate correlation (r = 0.30) with 90% power at an alpha level of 0.05. The sample size achieved exceeded this threshold, providing sufficient power (>90%) to detect statistically meaningful associations between variables. This sample size also supports generalizability across clinician specialties and regions within India [3,6,8].

Bias assessment

To assess potential common-method bias, we conducted Harman’s single-factor test. All Likert items were entered into an unrotated exploratory factor analysis. The first factor accounted for 76.02% of total variance, which exceeds the commonly cited 50% threshold, suggesting that common-method variance and/or halo effects may be present. Because this test is a coarse diagnostic, we interpreted associations cautiously and conducted sensitivity analyses using Spearman’s ρ [9].

Statistical analysis

Data were analyzed using IBM Corp. Released 2021. IBM SPSS Statistics for Windows, Version 27. Armonk, NY: IBM Corp. Descriptive statistics, including frequencies and percentages, were used to summarize clinician responses. We examined associations between patient-centric packaging items and overall therapy success using Pearson’s correlations with two-sided p-values. Although Likert-type responses are ordinal, prior work demonstrates that 5-point scales analyzed in sufficiently large samples yield approximately interval properties and that Pearson’s r is robust to such conditions [10-12]. Pearson’s correlation was chosen as it is widely used to assess relationships between continuous and ordinal variables in clinical research. To assess robustness, we conducted sensitivity analyses using Spearman’s rank correlation (ρ). Pearson 95% CIs were derived via Fisher’s z transform; Spearman 95% CIs were derived using a nonparametric bootstrap. Results from both approaches were directionally and substantively consistent. Statistical significance was defined as p < 0.05, in accordance with standard medical research practices.

## Results

Demographic characteristics of clinicians

Table 1 presents the demographic characteristics of the participating clinicians (N = 262). The sample was diverse in terms of specialty, years of clinical experience, and regional distribution across India. The majority of participants were consultant physicians (n = 186, 71.0%), followed by diabetologists (n = 32, 12.2%), cardiologists (n = 28, 10.7%), and endocrinologists (n = 13, 5.0%), with a small proportion from other specialties (n = 3, 1.1%). Regarding clinical experience, 102 (38.9%) had 11-20 years, followed by 97 (37.0%) with 10 years, 38 (14.5%) with 21-30 years, and 25 (9.5%) with over 30 years of experience. Geographically, the highest proportion of clinicians practiced in Western India, 95 (36.4%), and Northern India, 89 (34.1%), followed by Southern India, 68 (26.1%), while the Eastern region had the lowest representation, 9 (3.4%).

**Table 1:** Demographic characteristics of participating clinicians (N = 262).

Variable	Category	n	%
Specialty	Consultant Physicians	186	71.00%
	Diabetologists	32	12.20%
	Cardiologists	28	10.70%
	Endocrinologists	13	5.00%
	Others	3	1.10%
Years of Clinical Experience	≤10 years	97	37.00%
	11–20 years	102	38.90%
	21–30 years	38	14.50%
	>30 years	25	9.50%
Geographic Region	Western India	95	36.40%
	Northern India	89	34.00%
	Southern India	68	26.00%
	Eastern India	9	3.40%

Pill burden reduction and therapy simplification

**Figure 1:**
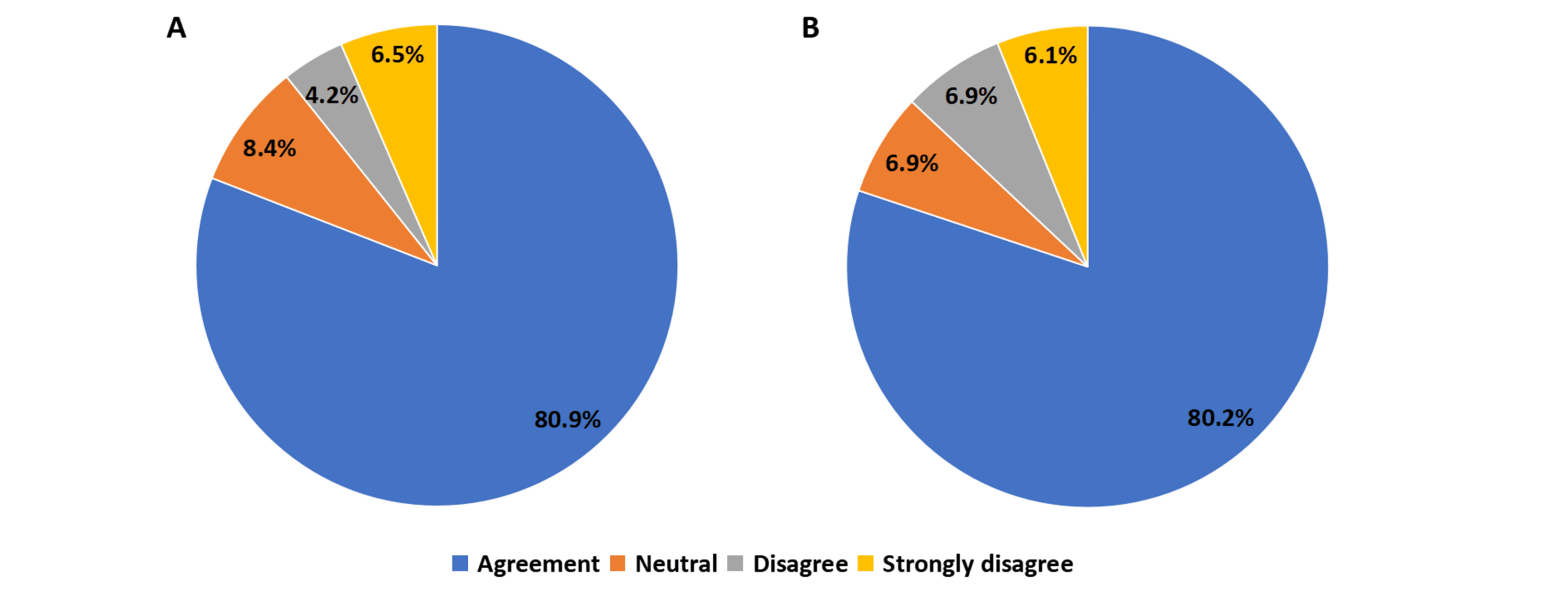
(A) Clinician-reported impact of the FDC on pill burden reduction and therapy simplification. (B) Clinician-reported perceptions of affordability as a factor in adherence. The image is created by the authors.

Among the respondents, 212 (80.9%) agreed that the FDC of dapagliflozin, glimepiride, and metformin significantly reduced pill burden and simplified therapy for patients (Figure 1A). Clinicians perceived that prescribing a single combination pill instead of three separate medications contributed to improved patient adherence and convenience. Additionally, 215 (82.1%) of respondents stated that the triple-drug FDC simplified therapy management, making it easier for patients to follow prescribed treatment regimens consistently.

Affordability as a driver of adherence

A total of 210 (80.2%) of clinicians perceived the triple-drug FDC as more affordable than prescribing the three medications separately, suggesting that the affordability of the combination therapy may contribute to better adherence (Figure 1B). While the majority favored the affordability of FDCs, 34 (13.0%) of respondents did not find a significant financial advantage, indicating that economic considerations may vary across clinical settings. Furthermore, 212 (80.9%) agreed that cost savings associated with the FDC directly influenced patient adherence.

These findings highlight the dual benefits of FDC therapy, reducing pill burden and enhancing affordability, both of which are critical for sustained medication adherence in diabetes care.

Visual appeal and ease of medication identification

**Figure 2:**
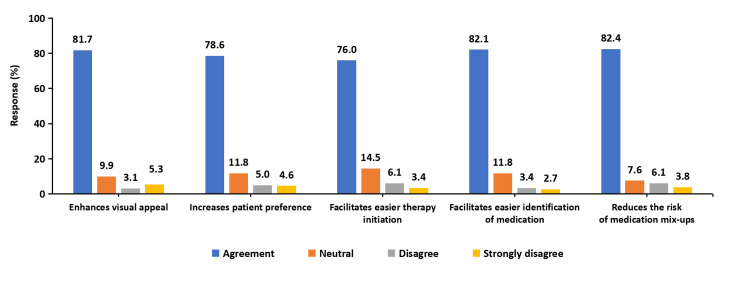
Clinician perspectives on the visual appeal and ease of use of the floral person-centric packaging (PCP) for the triple-drug FDC. The image is created by the authors.

**Figure 3:**
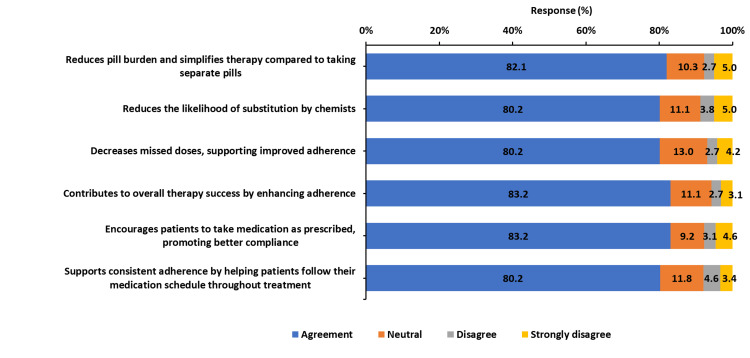
Impact of person-centric packaging (PCP) on adherence, therapy success, and medication substitution prevention for the triple-drug FDC. The image is created by the authors.

The PCP of the FDC was reported to enhance medication identification and patient preference. A total of 214 (82.0%) of clinicians agreed that the floral gold design improved medication recognition, making it easier for patients to distinguish their prescribed medication from other drugs (Figure 2). Additionally, 206 (78.6%) of respondents stated that PCP increased patient preference, with clinicians observing greater acceptance and engagement with the medication compared to traditional packaging. Clinicians also reported a notable reduction in pharmacy-driven substitutions due to the unique PCP design, with 210 (80.1%) agreeing that distinctive packaging reduced instances of unintended medication switching at the pharmacy level (Figure 2). Furthermore, 210 (80.2%) of clinicians noted that the improved packaging helped decrease missed doses, reinforcing its role in supporting adherence. Clinicians reported a strong association between PCP-integrated FDC and improved adherence, with 218 (83.2%) agreeing that the packaging played a role in encouraging patients to take their medication as prescribed (Figure 3). Additionally, 218 (82.5%) of respondents indicated that the packaging design contributed to overall therapy success by promoting better adherence and reducing instances of missed doses. A total of 200 (76.3%) of clinicians observed an increase in follow-up appointments among patients prescribed the PCP-integrated FDC. This suggests that the engaging and structured design of the packaging may have reinforced treatment continuity by encouraging patients to return for regular consultations.

Preference for PCP over traditional packaging

**Figure 4:**
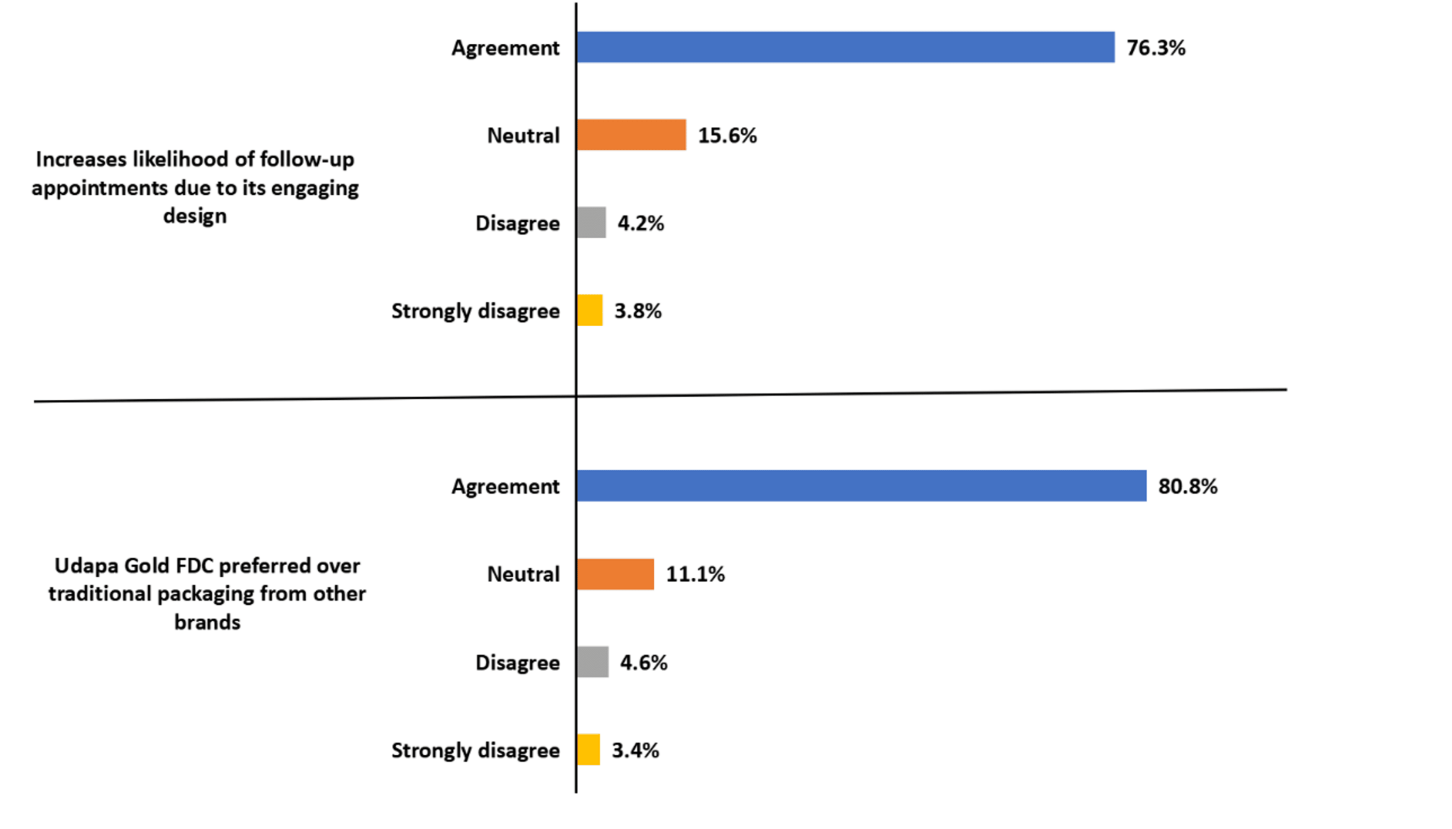
Clinician-reported preference for PCP over traditional packaging and its impact on follow-up engagement.

Clinicians expressed a strong preference for PCP over traditional pharmaceutical packaging. A total of 211 (80.8%) of respondents preferred the floral PCP design compared to conventional packaging from other brands, citing improved medication identification and enhanced patient engagement (Figure 4). These findings suggest that packaging innovations may influence not only adherence but also patient-clinician interactions, enabling continuous engagement with therapy.

Correlation between affordability, pill burden reduction, and adherence outcomes

The Pearson correlation analysis demonstrated significant positive relationships between the affordability of the triple FDC and various factors influencing medication adherence, therapy success, and patient engagement (Table 2). A strong correlation was observed between pill burden reduction and therapy simplification (r = 0.883, p < 0.001), highlighting the role of FDCs in enhancing treatment convenience. Additionally, the perception that the FDC contributes to overall therapy success was positively associated with adherence improvement (r = 0.720, p < 0.001). While statistically significant, these correlations may be partially influenced by shared method variance and should be interpreted with caution. The affordability of the FDC also showed a significant correlation with key adherence parameters, including encouraging patients to take medication as prescribed (r = 0.698, p < 0.001) and supporting consistent adherence (r = 0.700, p < 0.001).

**Table 2:** Pearson correlation analysis of PCP, affordability, adherence, and therapy success factors. FDC: Fixed-Dose Combination; r: Pearson Correlation Coefficient

Primary Factor	Associated Outcome	r	p-value
Affordability of the triple FDC of dapagliflozin, glimepiride, and metformin	Facilitates easier therapy initiation	0.677	<0.001
	Triple FDC of dapagliflozin, glimepiride, and metformin reduces pill burden and simplifies therapy	0.883	<0.001
	Contributes to overall therapy success by enhancing adherence	0.720	<0.001
	Encourages patients to take medication as prescribed	0.698	<0.001
	Supports consistent adherence by helping patients follow their medication	0.700	<0.001
	Increases likelihood of follow-up appointments	0.628	<0.001
Triple FDC reduces pill burden & simplifies therapy	Enhances visual appeal and ease medication identification	0.708	<0.001
	Increases preference due to visually appealing design	0.667	<0.001
	Facilitates easier therapy initiation	0.650	<0.001
	Facilitates easier identification	0.732	<0.001
	Reduces the risk of medication mix-ups	0.712	<0.001
	Decreases missed doses	0.693	<0.001
	Contributes to overall therapy success by enhancing adherence	0.717	<0.001
	Encourages patients to take medication as prescribed	0.687	<0.001
	Supports consistent adherence by helping patients follow their medication	0.695	<0.001
	Increases likelihood of follow-up appointments	0.601	<0.001

Further relationships were observed between pill burden reduction and factors such as medication identification (r = 0.732, p < 0.001), decreased risk of medication mix-ups (r = 0.712, p < 0.001), and fewer missed doses (r = 0.693, p < 0.001). Additionally, increased patient preference for PCP (r = 0.667, p < 0.001) and higher likelihood of follow-up appointments (r = 0.601, p < 0.001) were also reported.

Spearman’s correlation analysis demonstrated strong positive associations between pill burden reduction and multiple adherence-related outcomes. Pill burden reduction was most strongly correlated with adherence improvement (ρ = 0.881, p < 0.001) and therapy simplification (ρ = 0.870, p < 0.001), followed by medication identification (ρ = 0.728, p < 0.001) and fewer missed doses (ρ = 0.701, p < 0.001). Affordability was also positively correlated with adherence improvement (ρ = 0.693, p < 0.001) and therapy success (ρ = 0.685, p < 0.001). Similarly, PCP design showed positive correlations with patient preference (ρ = 0.661, p < 0.001), reduced pharmacy substitution (ρ = 0.642, p < 0.001), and follow-up visits (ρ = 0.589, p < 0.001) (Appendix, Table 2).

Appendix, Table 3 summarizes key findings from the clinician survey on FDC therapy and PCP in diabetes.

Exploratory subgroup analyses were conducted to examine whether perceptions of PCP varied by clinician specialty, region, or years of experience. While no statistically significant differences emerged, some trends were noted, for example, clinicians practicing in western India reported slightly higher perceived adherence benefits than those in other regions.

## Discussion

Medication packaging has traditionally been treated as an afterthought in drug development, finalized only once formulation and dosing are in place [13]. However, in chronic diseases like T2DM, where long-term adherence is essential, packaging may play an important supporting role in adherence. In this study, we evaluated how person-centric packaging (PCP), when used with a triple fixed-dose combination (FDC) of dapagliflozin, glimepiride, and metformin, impacted adherence and patient engagement. Our findings reflect strong clinician support for PCP, particularly in its ability to reduce pill burden, simplify therapy, and make treatment easier to follow. The role of FDCs in improving adherence is well documented. Previous studies show that FDCs reduce the number of pills and simplify dosing schedules, which can encourage patients to stay on treatment [3,14]. Our study aligns with this evidence, with over 80% of clinicians agreeing that the FDC reduced pill burden and simplified therapy. Importantly, 83.2% also felt that packaging played a role in improving adherence, supporting earlier findings on the benefits of structured packaging [8,15]. Previous systematic reviews have demonstrated that packaging design features, particularly calendar-based reminder systems, can significantly improve medication adherence in patients requiring long-term therapy [1,8].

Despite these benefits, poor packaging can still be a barrier to adherence. Similar-looking pills, unclear instructions, and generic packaging increase the risk of medication errors and confusion, especially for elderly patients or those managing multiple prescriptions [5,4]. Research shows that better design, such as clear labels, color codes, and structured layouts, can improve medication recognition and boost patient confidence [6]. PCP address these challenges by leveraging visual and structural cues in packaging, an approach supported by consumer neuroscience, which shows that multisensory packaging design can influence perception, recognition, and behavioral engagement with products [16]. In our study, over 80% of clinicians said that the floral PCP design helped patients identify their medication more easily, and nearly the same proportion reported fewer chemist substitutions. These results echo prior studies that suggest PCP may reduce the likelihood of pharmacist-driven medication switching by making branded formulations more visually distinct [6]. This could be especially relevant in systems where generics are frequently dispensed and substitution is common.

Economically, FDCs also offer value. A large proportion of clinicians in this study cited affordability as a reason why patients stayed on therapy, consistent with past research showing that FDCs lower treatment costs and improve outcomes [17]. Although our study captured perceptions rather than actual cost data, the consistent agreement across respondents suggests that reduced financial burden is a meaningful contributor to adherence in clinical practice.

The connection between packaging and patient behavior extends beyond adherence. A well-designed package can increase patient preference, improve follow-up rates, and support long-term engagement with treatment [18]. In our findings, clinicians noted improved patient interest and higher follow-up visit rates for those on the PCP-integrated FDC. Other studies have also found that patients associate visually appealing packaging with better quality and trustworthiness [19].

Overall, this study adds to the growing body of evidence that both clinical and non-clinical factors influence adherence. While medications must be effective and affordable, how they are delivered, both in therapy and experience, matters greatly. PCP and FDCs address two sides of the adherence equation: one simplifies the treatment regimen, and the other enhances how patients interact with it. Real-world care models like pharmacist-led collaborative management programs have shown sustained improvements in glycemic control and lower increases in medical costs [20]. In our study, too, a majority of clinicians preferred PCP-integrated FDCs over conventional packaging, citing ease of use and better patient acceptance. Importantly, these findings represent prescriber perceptions, not direct adherence data from patients. Therefore, the observed associations should be interpreted cautiously and not assumed to reflect causality.

While our findings suggest that clinicians perceive PCP as supportive of adherence, these data reflect perceptions rather than direct patient adherence measurements. PCP should therefore be interpreted as a complementary, low-burden intervention rather than a standalone solution. Compared with digital adherence tools (e.g., SMS prompts, smartphone apps, smart blister packs), PCP imposes minimal ongoing effort, requires no connectivity or digital literacy, and can be universally applied at the point of dispensing, but it lacks the real‑time feedback, remote monitoring, and personalized escalation that digital tools enable. Relative to education-based strategies, PCP may enhance salience and habit formation through visual cues and simplified organization, yet it does not substitute for tailored counseling, motivational interviewing, or health literacy interventions that address beliefs and self‑efficacy. In practice, PCP may be most effective as part of a multi-component approach (packaging + brief education + selective digital reminders), where packaging reduces friction and cognitive load, while other modalities address behavioral barriers and enable follow‑up [6,13]. Future studies should compare PCP head‑to‑head and in combination with digital and educational strategies using objective adherence endpoints (e.g., pharmacy refill data, electronic monitoring) and clinical outcomes.

Although the correlation between pill burden reduction and adherence was strong (r = 0.883), we recognize that methodological factors such as common-method variance and halo effects could have contributed to this result. Prior research in survey methodology [9,21] has emphasized the importance of multi-method assessment. We recommend that future studies adopt multi-source data collection strategies, introduce temporal separation between measurements, and consider statistical techniques such as marker variables or confirmatory factor analysis to further assess and control common-method bias. Additionally, the cross-sectional design limits causal inference; although significant associations were noted between PCP, adherence, and therapy success, longitudinal studies are essential to validate sustained benefits over time, especially as improving adherence may have a far greater impact on population health than advancements in treatment alone [22]. Beyond individual benefits, better adherence can lead to fewer complications, reduced hospital visits, and long-term cost savings for the healthcare system [23]. As healthcare systems shift toward more patient-focused approaches, combining FDCs with thoughtful, person-friendly packaging could be a simple yet powerful step toward improving outcomes in chronic disease management.

Limitations

Despite the valuable insights provided by this study, certain limitations should be acknowledged. First, the study relied on clinician-reported perceptions, which may introduce response bias due to individual experiences and subjective assessments. This study relied solely on clinician perceptions without patient-reported outcomes or objective adherence data. While the findings highlight strong support for PCP and FDCs in diabetes management, patient-reported outcomes were not collected, limiting the ability to directly assess adherence behavior from the end-user perspective [24]. Additionally, the cross-sectional design limits causal inference; although significant associations were noted between PCP, adherence, and therapy success, longitudinal studies are essential to validate sustained benefits over time [25]. Convenience sampling limits generalizability, and pre-survey meetings may have introduced priming bias. The educational briefings conducted before survey administration may have contributed to priming bias by emphasizing the benefits of the FDC and its packaging design. This may have influenced clinicians’ perceptions and represents a potential limitation. Restricting the sample to current prescribers may have introduced selection bias, as clinicians with less favorable views may have been excluded. Conducting multiple correlation analyses increases the potential for Type I error, and findings should be interpreted accordingly. Other potential limitations include social desirability, recall, and confirmation biases. Another limitation is that assessments of affordability were based on clinician perspectives, and further economic evaluations using real-world prescription data and healthcare utilization metrics would strengthen the evidence supporting the financial impact of PCP-integrated FDCs. Furthermore, the high correlation coefficients observed in this study (particularly r = 0.883 between pill burden reduction and improved adherence) may reflect common-method bias, as all data were collected via the same survey instrument administered to the same set of respondents. The single-survey format also increases the risk of common-method bias. Given the use of a single-source, self-report survey instrument, common-method variance may have contributed to the high correlation values observed. In addition, a halo effect may have occurred, wherein clinicians’ positive impressions of one attribute (e.g., visual design) influenced ratings of other aspects (e.g., perceived adherence impact). These artifacts could artificially inflate the strength of observed associations. Although Harman’s single-factor test did not indicate substantial common-method bias, this possibility cannot be entirely ruled out. Another limitation is that although exploratory subgroup analyses were conducted, the study was not powered for formal multivariable modeling. The reliance on descriptive and bivariate statistics limits the ability to control for potential confounders or interaction effects. Future research should consider using multiple data collection methods, such as combining clinician surveys with objective adherence measures (e.g., pharmacy refill data or electronic medication monitors) and incorporating separate patient-reported outcomes, to reduce the risk of common-method bias and enhance the robustness of findings. In addition, randomized sampling and patient-level data should be incorporated. Finally, future studies should incorporate multivariable regression or stratified modeling to better understand which clinician- or practice-level factors may influence perceptions of PCP efficacy.

Future research directions and practical implications

This study highlights the potential benefits of PCP in FDC therapies, particularly in diabetes management. Future research should focus on real-world patient-reported adherence data to validate findings beyond clinician perceptions. Longitudinal studies assessing the impact of medication adherence in diabetes care demonstrate that improved adherence significantly reduces inpatient costs and enhances clinical outcomes, supporting the need for sustained adherence-focused interventions [26]. Additionally, qualitative research on patient perspectives regarding packaging usability, preference, and behavioral adherence mechanisms could offer deeper insights into how visual and structural design elements influence medication-taking behavior [13].

Further research should explore health economic evaluations quantifying cost savings from improved adherence, reduced hospitalizations, and fewer diabetes-related complications. Integrating digital adherence tools, such as NFC-enabled smart packaging or app-based reminders, could complement PCP innovations and offer personalized adherence interventions [18]. Policymakers and pharmaceutical stakeholders should explore scalable, affordable PCP solutions that align with regulatory guidelines while prioritizing patient-centric approaches in medication design.

## Conclusions

This study demonstrates the significant benefits of the triple FDC of dapagliflozin, glimepiride, and metformin in reducing pill burden, simplifying therapy, and improving medication adherence. Clinicians widely recognized affordability as a key driver of adherence, reinforcing the role of FDCs in optimizing long-term treatment outcomes. Additionally, PCP, with its floral gold design, was highly valued for enhancing medication identification, increasing patient preference, and improving therapy satisfaction. The strong correlations between affordability, pill burden reduction, therapy simplification, and adherence further highlight the importance of packaging innovations in diabetes management.

Future research should focus on real-world patient-reported adherence outcomes, longitudinal assessments of therapy persistence, and economic evaluations to quantify healthcare cost savings associated with PCP-integrated FDCs. Additionally, exploring PCP applicability in other chronic diseases could provide insights into its broader impact on medication adherence and patient engagement. As healthcare moves toward patient-centered models, integrating innovative packaging with pharmacological advancements presents a promising strategy to enhance treatment effectiveness and long-term disease management.

## Appendices

Table 3 shows the summary of questionnaire. Table 4 shows Spearman’s rank correlation (ρ) between key variables in the GOLD-PCP study. Table 5 shows the clinical survey.

**Table 3:** Study questionnaire. This is an original creation by the authors.

Questionnaire: Evaluating the Impact of Person-Centric Packaging on Medication Adherence in Diabetes Management		
Sl. No.	Question	Options
1	I have read the survey brief and agree to participate in this study with the understanding that my responses will remain anonymous and used solely for research purposes.	• Yes • No
2	Specialty	• General Physician (MBBS) • Consultant Physician (MD/DNB) • Endocrinologist (DM/DNB/MRCP) • Diabetologist (D Diab, Fellowship etc.) • Cardiologist (DM/DNB/MRCP) • Others
3	Years of experience	• 10 years or less • 11–20 years • 21–30 years • >30 Years
4	State of practice	(Open-ended)
5	The 3 drug FDC (dapagliflozin, glimepiride, metformin) in Udapa Gold reduces pill burden and simplifies therapy compared to taking separate pills.	Scale: 1 - Strongly Disagree to 5 - Strongly Agree
6	The cost-effectiveness of Udapa Gold’s FDC, compared to separate medications, is a key factor in improving patient adherence	Scale: 1 - Strongly Disagree to 5 - Strongly Agree
7	Enhances visual appeal and ease of medication identification for patients.	Scale: 1 - Strongly Disagree to 5 - Strongly Agree
8	Increases patient preference due to its visually appealing design.	Scale: 1 - Strongly Disagree to 5 - Strongly Agree
9	Facilitates easier therapy initiation, as patients are more willing to start treatment.	Scale: 1 - Strongly Disagree to 5 - Strongly Agree
10	Facilitates easier identification of medication compared to traditional packaging.	Scale: 1 - Strongly Disagree to 5 - Strongly Agree
11	Reduces the risk of medication mix-ups, especially for elderly or polypharmacy patients.	Scale: 1 - Strongly Disagree to 5 - Strongly Agree
12	Reduces pill burden and simplifies therapy compared to taking separate pills.	Scale: 1 - Strongly Disagree to 5 - Strongly Agree
13	Reduces the likelihood of substitution by chemists.	Scale: 1 - Strongly Disagree to 5 - Strongly Agree
14	Decreases missed doses, supporting improved adherence.	Scale: 1 - Strongly Disagree to 5 - Strongly Agree
15	Contributes to overall therapy success by enhancing adherence.	Scale: 1 - Strongly Disagree to 5 - Strongly Agree
16	Encourages patients to take medication as prescribed, promoting better compliance.	Scale: 1 - Strongly Disagree to 5 - Strongly Agree
17	Supports consistent adherence by helping patients follow their medication schedule throughout treatment.	Scale: 1 - Strongly Disagree to 5 - Strongly Agree
18	Increases likelihood of follow-up appointments due to its engaging design.	Scale: 1 - Strongly Disagree to 5 - Strongly Agree
19	Preferred over traditional packaging from other brands in enhancing patient satisfaction and adherence.	Scale: 1 - Strongly Disagree to 5 - Strongly Agree
20	What additional packaging features could further improve patient adherence and experience in chronic therapy?	(Open-ended)
21	Which other therapies do you believe could benefit from this patient-centric packaging design?	(Open-ended)

**Table 4:** Spearman’s rank correlation (ρ) between key variables in the GOLD-PCP study (n = 262). PCP: person-centric packaging. Note: All correlations were statistically significant at p <0.001, Results from Spearman’s rank correlation was consistent with those obtained using Pearson’s correlation, confirming the robustness of findings despite the ordinal nature of Likert-type scales.

Parameter 1	Parameter 2	Spearman’s ρ	p-value
Pill burden reduction	Adherence improvement	0.881	<0.001
	Therapy simplification	0.870	<0.001
	Medication identification	0.728	<0.001
	Fewer missed doses	0.701	<0.001
Affordability	Adherence improvement	0.693	<0.001
	Therapy success	0.685	<0.001
PCP design	Patient preference	0.661	<0.001
	Reduced pharmacy substitution	0.642	<0.001
	Follow-up visits	0.589	<0.001

**Table 5:** Clinician survey on FDC therapy and PCP in diabetes.

Impact on medication adherence and therapy simplification
The triple-drug FDC (dapagliflozin, glimepiride, metformin) reduces pill burden and simplifies therapy compared to taking separate pills. The affordability of the FDC, compared to separate medications, is a key factor in improving patient adherence. The FDC contributes to overall therapy success by enhancing medication adherence and simplifying treatment regimens.
Role of packaging in medication identification and patient engagement
The person-centric packaging (PCP) with a visually distinctive floral gold design enhances medication identification for patients. The visually appealing design increases patient preference and satisfaction compared to traditional packaging. Facilitates easier medication recognition, reducing the likelihood of confusion, especially for elderly and polypharmacy patients.
Prevention of medication errors and chemist substitutions
Reduces the risk of medication mix-ups, particularly in elderly patients and those on multiple medications. Decreases the likelihood of substitution by chemists, ensuring that patients receive the intended medication.
Support for treatment adherence and follow-up engagement
Encourages patients to take medication as prescribed, promoting better compliance and sustained adherence. Supports consistent adherence by helping patients follow their medication schedule throughout treatment. The engaging packaging design increases the likelihood of follow-up appointments, reinforcing long-term treatment engagement. Clinicians prefer the PCP-integrated FDC over traditional packaging from other brands for its impact on patient satisfaction, adherence, and therapy success.
